# Effect of Red Ginseng Extract on the Pharmacokinetics and Efficacy of Metformin in Streptozotocin-Induced Diabetic Rats

**DOI:** 10.3390/pharmaceutics10030080

**Published:** 2018-07-03

**Authors:** So Jeong Nam, You Jin Han, Wonpyo Lee, Bitna Kang, Min-Koo Choi, Yong-Hae Han, Im-Sook Song

**Affiliations:** 1 College of Pharmacy and Research Institute of Pharmaceutical Sciences, Kyungpook National University, Daegu 41566, Korea; goddns159@nate.com (S.J.N.); gksdbwls2@nate.com (Y.J.H.); 2 College of Pharmacy, Dankook University, Cheon 31116, Korea; dldjsvy93@naver.com (W.L.); qlcska@gmail.com (B.K.); minkoochoi@dankook.ac.kr (M.-K.C.); 3 Life Science Institute, Daewoong Pharmaceutical, Yongin 17028, Korea; yonghae.han@gmail.com

**Keywords:** Korean red ginseng extract, metformin, diabetes, drug interaction, pharmacokinetics, efficacy

## Abstract

The purpose of this study was to investigate the effect of red ginseng extract on the pharmacokinetics (PK) and efficacy of metformin in streptozotocin-induced diabetic rats. The diabetes mellitus rat model was established by intraperitoneally administering multiple doses of streptozotocin (30 mg/kg, twice on day 1 and 8), and diabetic rats received metformin 50 mg/kg with or without single or multiple administration of Korean red ginseng extract (RGE, 2 g/kg/day, once or for 1 week). RGE administration did not affect the plasma concentration and renal excretion of metformin. Further, diabetic rats were administered metformin (50 mg/kg) and RGE (2 g/kg) alone or concomitantly for 5 weeks, and both regimens decreased the fasting blood glucose and glycated hemoglobin (Hb-A1c) levels. Furthermore, fasting blood glucose levels were reduced by metformin or RGE administered alone but recovered to the control level following co-administration, suggesting that the effect was additive. However, triglyceride and free fatty acid levels were not different with metformin and RGE treatment alone or in combination. Biochemical parameters such as alanine aminotransferase (ALT), aspartate aminotransferase (AST), triglycerides, total cholesterol, high-density lipoprotein (HDL) cholesterol, low-density lipoprotein (LDL) cholesterol levels were not different among the three treatment groups. In conclusion, RGE and metformin showed an additive effect in glycemic control. However, the co-administration of RGE and metformin did not cause PK interactions or affect biochemical parameters including the free fatty acid, triglyceride, AST, ALT, or cholesterol levels.

## 1. Introduction

Diabetes mellitus (DM) is a common chronic disease worldwide and is major health problem [1,2]. Type 2 DM accounts for approximately 90% of all incidences of DM and is characterized by insulin resistance and pancreatic β-cell dysfunction [2,3,4,5]. In patients with type 2 DM, metformin has been recommended as a first-line treatment. Metformin lowers blood glucose concentration without increasing insulin secretion [6,7,8]. However, metformin alone is thought to be insufficient for glycemic control and, therefore, often requires co-therapy with other agents. Thus, drug–drug interactions between co-therapy drugs in patients with type 2 DM should be carefully considered and monitored. Red ginseng is obtained from fresh ginseng grown for 6 years through the process of steaming and drying, and has been reported to provide various therapeutic effects for diseases including cardiovascular disease, diabetes, allergies, insomnia, gastritis, hepatotoxicity, and sexual dysfunction [9,10,11]. Korean red ginseng was beneficial in a diabetic mouse model induced by streptozotocin (STZ) administration, not only for its hypoglycemic effects but also for its immunomodulation [12]. Supplementation with Korean red ginseng or Korean red ginseng extract (RGE) has been shown to improve DM in STZ-induced diabetic animals as well as in humans [12,13,14,15]. Fermented red ginseng also exhibited a strong antidiabetic effects in STZ-induced diabetic rats [12] and patients with type 2 DM [13]. STZ is most commonly used to establish experimental DM models because it damages pancreatic β-cells of the islets of Langerhans [16]. High-dose STZ severely impairs insulin secretion, similar to type 1 DM, but low-dose STZ causes some damage to insulin secretion, similar to type 2 DM. STZ can also be administered in multiple low doses to gradually achieve immune destruction of β-cells [3]. Consequently, we established the DM rat model used in this study with multiple low doses of STZ (two 30 mg/kg intraperitoneal injections administered 1 week apart) according to the method of Zhang et al. [2,3]. Using this STZ-induced DM rat model, therefore, the purpose of this study was to evaluate the possibility of drug–drug interactions between RGE and metformin based on their PK and efficacy.

## 2. Materials and Methods

### 2.1. Materials

Korean RGE was obtained from Punggi Ginseng Cooperative Association (Punggi, Korea), and was produced in the facilities following the current guidelines of the Korea Good Manufacturing Practice (Lot No. 1614-2). Furthermore, 2 g RGE contained 3.85 mg ginsenoside Rb1, 1.85 mg ginsenoside Rb2, 2.0 mg ginsenoside Rd, and 2.6 mg ginsenoside Rg3. Metformin and STZ were purchased from Sigma-Aldrich Corp. (St. Louis, MO, USA). All other reagents and solvents were of reagent grade.

### 2.2. Animals

Male Sprague−Dawley rats (7–8 weeks, 220−250 g) were purchased from Samtako Co. (Osan, Korea). On arrival, the rats were housed on a 12-h light/dark cycle and were provided food and water ad libitum for 1 week prior to the animal experiments. All animal procedures were approved by the Animal Care and Use Committee of Kyungpook National University (Approval No. 2017-0021) and carried out in accordance with the National Institutes of Health guidance for the care and the use of laboratory animals.

### 2.3. Induction of Diabetes Mellitus (DM) and Efficacy Monitoring

The rats were fasted overnight before the induction of DM with STZ. The rats were administered STZ 30 mg/kg (dissolved in 0.1 M citrate buffer, pH 4.5) intraperitoneally once and the treated was repeated 1 week later. Rats were fed for 4 h post STZ dose to avoid the anticipated hypoglycemic shock. Body weights, water intake, and urine output were closely monitored daily after STZ administration. Fasting blood glucose concentration was measured in tail vein blood using an Accu-Chek glucometer (Roche Korea, Seoul, Korea), every 2 or 3 days at 9 a.m. following overnight fasting for 35 days. Rats with fasting blood glucose >250 mg/dL were considered diabetes-induced and used in this study.

STZ-induced diabetic rats were divided into four groups: DM control (DC), DM with metformin (D + M), DM with RGE (D + RGE), and DM with metformin and RGE groups (D + RGE + M). Oral administration of water (vehicle), metformin, and red ginseng to rats were conducted daily via oral gavage.

Rats in the DC group were orally administered water as the vehicle for 5 weeks in addition to STZ administration. Rats in the D + M group received water as the vehicle and 2 h later they were orally administered metformin (50 mg/kg, dissolved in water). Rats in the D + RGE group received RGE (2 g/kg, dissolved in water) orally. Rats in the D + GRE + M group first received RGE (2 g/kg, dissolved in water) and 2 h later, metformin (50 mg/kg, dissolved in water) orally. At the end of the experimental day, abdominal arterial blood and the liver and pancreatic tissues were collected from rats in all groups. The collected blood was centrifuged at 13,000 rpm for 10 min at 4 °C and the supernatant plasma samples were used to the biochemical parameters such as alanine aminotransferase (ALT), aspartate aminotransferase (AST), triglycerides, total cholesterol, high-density lipoprotein (HDL)-cholesterol, low-density lipoprotein (LDL)-cholesterol, free fatty acids, and hemoglobin A1c (Hb-A1c). These biochemical parameters were measured in Seoul Clinical Laboratories (Yongin, Korea).

### 2.4. Pharmacokinetic Interaction Study

STZ-induced diabetic rats were divided into three groups: control, single administration of RGE (SA), and repeated administrations of RGE for 1 week (1WRA). The rats were fasted for at least 12 h before the oral administration of metformin.

Rats in the SA group were administered RGE (2 g/kg, 2 mL/kg suspended in water) once and 2 h later, they received metformin (50 mg/kg, dissolved in water) or vehicle orally. Rats in the 1WRA group received RGE suspension (2 g/kg/day, 2 mL/kg suspended in water) orally at 9 a.m. for 7 days. Twenty four hours after the last dose of RGE, rats received metformin (50 mg/kg, dissolved in water) or vehicle orally. For the comparison (control group), rats in were administered water as the vehicle for 8 days and 2 h later, they received metformin (50 mg/kg, dissolved in water) orally. Rats were kept in a metabolic cage to collect the urine during the experimental procedure. Blood samples were collected at 0, 0.083, 0.25, 0.5, 1, 2, 3, 4, 8, and 24 h via the retro-orbital vein following oral administration of metformin. The blood was centrifuged at 13,200 rpm for 10 min to separate the plasma. Urine samples were collected for 24 h. Aliquots (50 μL) of plasma and urine samples were stored at −80 °C until the analysis.

### 2.5. LC-MS/MS Analysis of Metformin and Ginsenoside Rb1

The concentration of metformin was analyzed using a modified liquid chromatography-tandem mass spectrometry (LC-MS/MS) method as previously reported by Kwon et al. [17]. Briefly, plasma and urine samples (50 μL) were mixed with 100 μL propranolol (internal standard, IS) in acetonitrile using a vortex mixer for 2 min. After centrifugation at 13,200 rpm for 5 min, an aliquot (2 μL) was injected into the Agilent 6430 Triple Quad LC-MS/MS system (Agilent, Wilmington, DE, USA), which was coupled to an Agilent 1260 series high-performance liquid chromatography (HPLC) system. The separation was performed using a Synergy Polar reverse phase (RP) column (2.0 mm × 150 mm, 4 µm particle size, Phenomenex, Torrence, CA, USA) using a mobile phase that consisted of methanol and water (70:30, *v*/*v*) with 0.1% formic acid at a flow rate of 0.2 mL/min. The retention time was 2.09 and 3.03 min for metformin and propranolol (IS), respectively. The mass spectra were recorded using electrospray ionization in a positive mode. Quantification was carried out using selected reaction monitoring at *m*/*z* 130.2 → 71.4 for metformin, and *m*/*z* 260.0 → 116.0 for propranolol (IS). Plasma and urine calibration standards were 0.1–15 μg/mL. The interday precision and accuracy were within the acceptance criteria for assay validation.

The plasma Rb1 concentration was analyzed using an Agilent 6470 Triple Quadrupole LC MS/MS system with a modified method of Choi et al. [18]. Briefly, plasma samples (50 μL) were mixed with 200 μL of methanol containing berberine (0.5 ng/mL; IS) for 10 min and centrifuged. An aliquot (10 μL) of the supernatant was injected into the LC-MS/MS system. Separation was performed on a Synergi Polar RP column using a mobile phase consisting of water and methanol (24:76, *v*/*v*) with 0.1% formic acid at a flow rate of 0.2 mL/min. Quantification was carried out at *m*/*z* 1131.6 → 365.1 for Rb1 and *m*/*z* 336.1 → 320.0 for berberine (IS) in the positive ionization mode. For the analytical validation of Rb1 in plasma samples, the standard curve range was 0.5–100 ng/mL.

### 2.6. Data Analysis

PK parameters were calculated using the WinNonlin (version 2.0, Pharsight Corporation, Mountain View, CA, USA) using non-compartmental analysis. The data are expressed as the means ± standard deviation (SD) for the groups.

Statistical analysis was performed using the Student *t*-test (between two groups), one-way ANOVA test (among three groups), or two-way ANOVA test (differences between time period and treatment groups in fasting blood glucose level). In all cases, a difference was considered significant when *p* < 0.05.

## 3. Results

### 3.1. Pharmacokinetics (PK) Interaction between Metformin and Red Ginseng Extract (RGE) in Diabetic Rats

**Figure 1:**
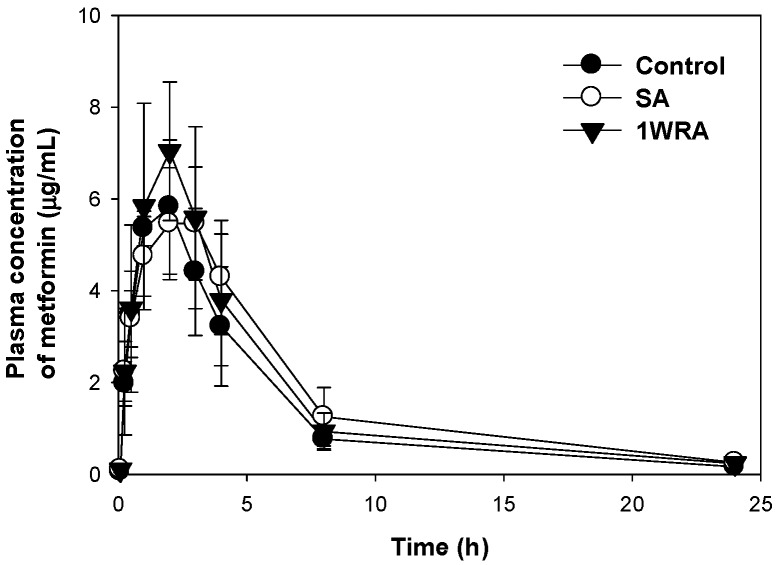
Plasma concentration-time profile of metformin after single oral administration (50 mg/kg) alone (●, control) and following single (○, SA) or multiple administration (▼, 1WRA) of red ginseng extract (RGE, 2 g/kg/day) in streptozotocin (STZ)-induced diabetic rats. Data points are means ± SD of five rats.

The PK profiles of metformin after a single oral administration of metformin (50 mg/kg) in the presence or absence of single or multiple administration of RGE (2 g/kg) are shown in Figure 1, and the PK parameters calculated from the plasma concentration-time profile are presented in Table 1. There was no significant difference in any PK parameters of metformin among the three groups. Moreover, the urinary recovery of metformin was not changed by the single or multiple administration of RGE. The results suggested that RGE pretreatment did not affect the absorption or disposition of metformin and, consequently, the plasma concentrations of metformin were not changed by co-administration of RGE.

**Figure 2:**
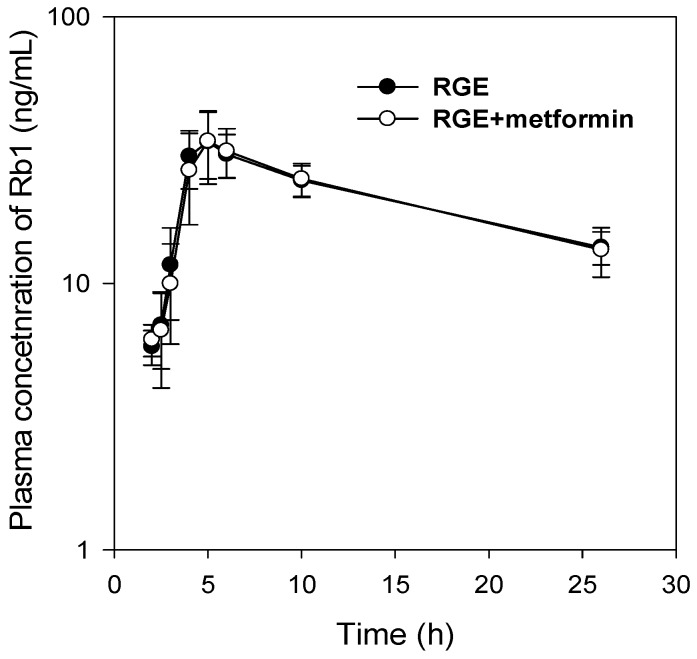
Plasma concentration–time profile of ginsenoside Rb1 following a single oral dose of RGE (2 g/kg) in the absence or presence of metformin (50 mg/kg) in streptozotocin (STZ)-induced diabetic rats. Data points are means ± SD of five rats.

Similarly, the PK profiles and all the PK parameters of ginsenoside Rb1 following a single oral dose (2 g/kg) of RGE was not changed by the co-administration of metformin (50 mg/kg) (Figure 2 and Table 2).

### 3.2. Efficacy of Metformin with RGE in Diabetic Rats

#### 3.2.1. Fasting Blood Glucose

**Figure 3:**
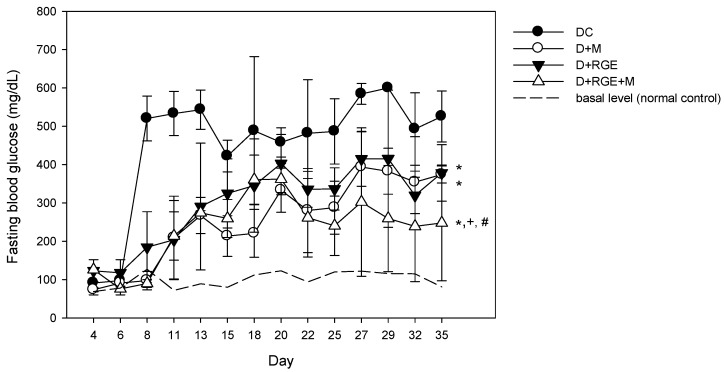
Effects of oral administration of metformin (M) and red ginseng extract (RGE) alone or combined on fasting blood glucose concentration of normal control (---), streptozotocin (STZ)-induced diabetic group (●, DC), diabetic rats treated with metformin (○, D + M, 50 mg/kg/day), diabetic rats supplemented with RGE (▼, D + RGE, 2 g/kg/day), and diabetic rats treated with metformin and RGE (∆, D + RGE + M). Data points are means ± SD of four different rats per group. * *p* < 0.05 compared with DC group; + *p* < 0.05 compared with D + M group; # *p* < 0.05 compared with D + RGE group using two-way ANOVA test.

The effect of oral administration of metformin, RGE, or their combinations on fasting blood glucose is shown in Figure 3. Fasting blood glucose was not elevated on day 4 after the first low dose STZ administration (30 mg/kg), but it increased to approximately 500 mg/dL after the second STZ administration (30 mg/kg) and a high fasting blood glucose level was maintained for more than 1 month. Metformin supplementation prevented the increase in fasting blood glucose level for 1 week but the second STZ administration gradually increased the value to approximately 200 mg/dL, and after 20 days of metformin administration (50 mg/kg for 5 weeks), the fasting glucose level increased to approximately 300–400 mg/dL. However, the fasting glucose levels for all periods were lower than that of the diabetic control group. RGE supplementation also gradually increased the fasting blood glucose for the 20-day supplementation and showed steady-state fasting glucose levels of approximately 300–400 mg/dL. Supplementation with metformin or RGE alone induced similar fasting blood glucose levels. The co-administration of metformin and RGE maintained the fasting blood glucose level at approximately 200 mg/dL, and it was significantly lower than that of the diabetic rats supplemented with metformin or RGE alone. The results suggest the additive effect of coadministration of RGE (2 g/kg) and metformin (50 mg/kg) for 5 weeks.

#### 3.2.2. Body, Liver, and Pancreas Weight

**Figure 4:**
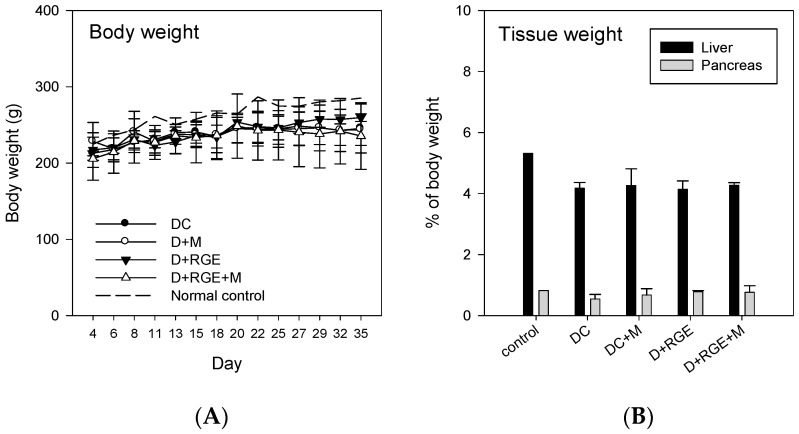
Effects of oral administration of metformin and red ginseng extract (RGE) alone or combined on body (**A**) and liver or pancreas (**B**) weights in normal control, streptozotocin (STZ)-induced diabetic group (DC), diabetic rats treated with metformin (D + M, 50 mg/kg/day), diabetic rats supplemented with RGE (D + RGE, 2 g/kg/day), and diabetic rats treated with metformin and RGE (D + RGE + M). Data points and bars are means ± SD of four rats per group.

There were no significant differences in the total body, liver, and pancreas tissue weights between treatment groups (Figure 4). However, the body, liver, and pancreas tissue weights of all treatment groups were lower than those of the normal control group.

#### 3.2.3. Biochemical Results

**Figure 5:**
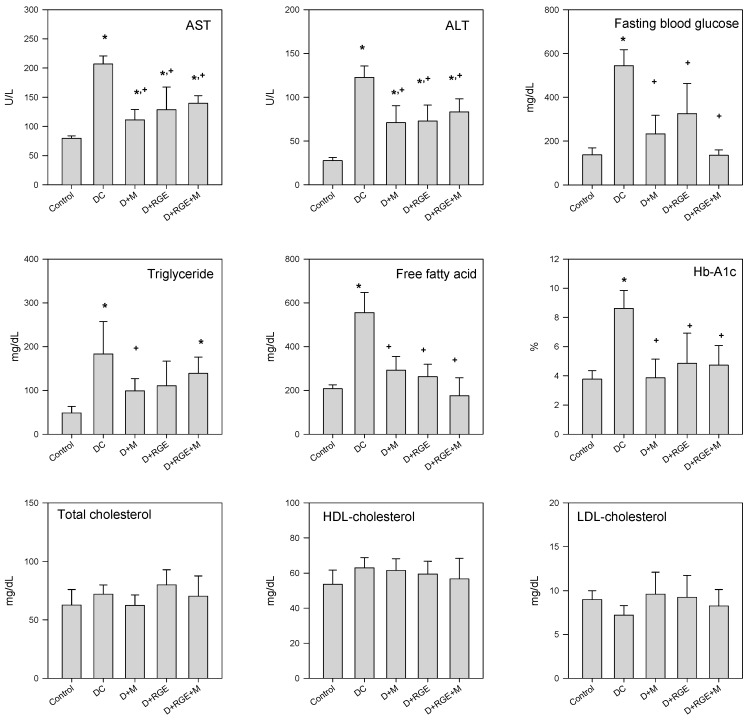
Biochemical parameters, alanine aminotransferase (ALT), aspartate aminotransferase (AST), fasting blood glucose, triglyceride, free fatty acid, hemoglobin-A1c (Hb-A1c), total cholesterol, high-density lipoprotein (HDL)-cholesterol, and low-density lipoprotein (LDL)-cholesterol levels in normal control, streptozotocin (STZ)-induced diabetic group (DC), diabetic rats treated with metformin (D + M), diabetic rats supplemented with RGE (D + RGE), and diabetic rats treated with metformin and RGE (D + RGE + M). Bars represent means ± SD of four rats per group; * *p* < 0.05 compared with control group using Student’s *t*-test; + *p* < 0.05 compared with DC group using Student’s *t*-test.

Figure 5 shows the plasma AST and ALT levels after administration of metformin and RGE alone or in combination for 35 days. The levels of both AST and ALT in the diabetic groups increased significantly compared with that of the control group but significantly decreased by metformin and RGE alone or co-administered; however, the levels did not return to control levels. Fasting glucose level results were similar to those shown in Figure 3. The HbA1c level of the DC group increased more significantly than that of the control group, and decreased to control levels following administration of metformin and RGE alone or in combination. Likewise, the levels of free fatty acid and triglyceride were also increased in the STZ-induced diabetic group but significantly lowered by metformin and RGE alone or co-administered. However, the levels of total, HDL-, and LDL-cholesterol showed no significant difference between the STZ-induced diabetic and metformin- or RGE-treated groups.

## 4. Discussion

STZ has been widely used to establish animal models of DM. In the present study, the administration of multiple low dose injections of STZ to rats was used to induce a mild impairment of insulin secretion and gradual autoimmune-like destruction of β-cells according to the method of Zhang et al. [14]. Moreover, they reported that STZ twice injection showed >85% success rate in development of DM, which was stable for >8 weeks [14]. In our study, the fasting glucose level was approximately 520 ± 58 mg/dL in six of the eight rats and was maintained for 4 weeks after the second STZ injection. 

Since the PK of metformin could differ between diabetic and normal rats, we aimed to investigate the PK drug interaction between metformin and RGE in diabetic rats. Moreover, REG suspension (2 g/kg in 2 mL of water) has been reported to contain ginsenosides, polysaccharides, fatty acids, peptides, and polyacetylenic alcohols [19], and, therefore, metformin was administered orally 2 h after pre-treatment with RGE suspension to avoid physical interaction of both substances considering that the average gastric emptying time in rats is approximately 30 min [20,21]. The results showed that the plasma concentration, absorption, and urinary excretion of metformin were not modulated by pre-treatment of RGE to diabetic rats, suggesting that the possibility of herb–drug PK interaction between metformin and RGE is remote.

The mechanisms underlying the modulation of glucose metabolism by RGE in patients with DM would likely be perturbation of hepatic glucose production and enhancement of glucose uptake through glucose transporter 4 (GLUT4) into peripheral tissues. Recently, ginsenosides Rb1, Rb2, Rg1, Rg3, Rh2, and compound K, major pharmacological components of RGE, have been reported to suppress the hepatic gluconeogenesis via adenosine monophosphate (AMP)-activated protein kinase (AMPK) [22,23,24]. Importantly, enhanced GLUT4 expression mediated by activated insulin receptor substrate (IRS)/phosphatidylinositol-3,4,5-triphosphate (PI3K)/serine-threonine protein kinase signaling pathway by the treatment of RGE or ginsenosides increased glucose uptake in adipocytes or skeletal muscle cells and, thereby, decreased blood glucose or Hb-A1c levels [22,25]. In addition, enhancement of glucose uptake via GLUT4 through the upregulation of adipocytic peroxisome proliferator-activated receptor-γ also contributes to the ginsenoside-mediated glucose control mechanism [26]. The molecular mechanisms underlying the actions of metformin appear to be related to its activation of AMPK, which suppresses glucagon-stimulated glucose production, and increases glucose uptake via GLUT4 in muscles and hepatic cells [27,28]. Because of the similarity between metformin and RGE, the effects of herb–drug interaction on their efficacy seem to be important.

The levels of fasting blood glucose seemed to indicate an additive effect of the co-administration of metformin and RGE because these levels were not recovered to the normal state by the treatment of metformin (50 mg·kg^−1^·day^−1^) or RGE (2 g·kg^−1^·day^−1^) alone. However, co-treatment with metformin and RGE significantly reduced the fasting blood glucose level compared with those of monotherapy with metformin or RGE (Figure 5). Moreover, the fasting blood glucose level, which was monitored for 5 weeks after the induction of DM, showed more stability and was in the range of 200 mg/dL following co-administration of metformin and RGE. Considering the lack of PK herb–drug interaction between metformin and RGE, the additive effect of metformin and RGE on glucose control could be attributed to their similar mechanisms of action. In addition to the glucose control in STZ-induced diabetic rats, interactions of biochemical parameters including the free fatty acid level, triglyceride, AST, ALT, or cholesterol levels were not shown by the co-administration of metformin and RGE. However, we should note that, in this study, we measured fasting blood glucose level and Hb-A1c as efficacy markers for metformin and RGE treatment but not included additional corroborative hypoglycemic efficacy markers such as oral glucose test, insulin level, GLUT4 levels in the liver or skeletal muscle, and other signaling pathway markers (AMPK and IRS/PI3K) to unveil the related mechanisms by metformin and RGE interaction.

## Figures and Tables

**Table 1:** Pharmacokinetic parameters of metformin after single oral administration (50 mg/kg) alone and following single (SA) or multiple administration (1WRA) of red ginseng extract (RGE, 2 g/kg/day) in streptozotocin (STZ)-induced diabetic rats.

Parameters		Control	SA	1WRA	*p* Value
C_max_	μg/mL	6.29 ± 1.04	5.54 ± 1.20	7.58 ± 1.39	0.06
T_max_	h	1.80 ± 0.45	2.40 ± 0.55	1.80 ± 0.84	0.26
AUC_24 h_	μg·h/mL	33.07 ± 6.82	41.59 ± 11.93	39.58 ± 9.50	0.37
AUC_∞_	μg·h/mL	34.27 ± 6.63	43.49 ± 11.72	41.18 ± 10.30	0.33
t_1/2_	h	4.59 ± 1.14	5.05 ± 0.64	4.53 ± 0.40	0.65
MRT	h	5.68 ± 1.28	6.63 ± 0.77	5.77 ± 0.91	0.29
Ae_24 h_	% of dose	45.14 ± 3.26	48.37 ± 5.87	41.89 ± 9.19	0.33

Data were expressed as mean ± SD from five rats of control, SA, and 1WRA groups, respectively. *p* value indicates static comparison among three groups using one-way ANOVA test. C_max_: maximum plasma concentration; T_max_: time to reach C_max_. AUC_24 h_ or AUC_∞_: Area under plasma concentration–time curve from zero to 24 h or infinity. t_1/2_: elimination half-life; MRT: mean residence time. Ae_24 h_: the fraction of the dose excreted in urine for 24 h as a parent form.

**Table 2:** Plasma concentration-time profile of ginsenoside Rb1 following a single oral dose (2 g/kg) of red ginseng extract (RGE)in the absence or presence of metformin (50 mg/kg) in streptozotocin (STZ)-induced diabetic rats.

Parameters		RGE	RGE + Metformin	*p* Value
C_max_	ng/mL	36.59 ± 7.94	35.03 ± 8.82	0.31
AUC_24 h_	ng·h/mL	512.25 ± 30.06	511.45 ± 75.26	0.98
t_1/2_	h	11.88 ± 0.75	11.86 ± 0.79	0.93

Data points are means ± SD of five rats. *p* value indicates static comparison between two groups using student *t*-test.
